# 

**Published:** 1875-11

**Authors:** 


FOUR DOLLARS A YEAR, POSTAGE FREE.

VOL. XXXII. November, 1875. NUMBER 11.

### THE Chicago Medical Journal AND EXAMINER.

S.G.O. INDEXED

ISSUED TWELVE TIMES A YEAR.

EDITOR:

WILLIAM H. BYFORD, A.M., M.D.

ASSOCIATE EDITORS:

JAS. H. ETHERIDGE, M.D.

NORMAN BRIDGE, M.D.

JAS. NEVINS HYDE, A.M., M.D.

FERD. C. HOTZ, M.D.

TERMS—FOUR DOLLARS PER ANNUM, IN ADVANCE.

CHICAGO:

W. B. KEEN, COOKE & CO., Publishers,

NOS. 113 AND 115 STATE STREET.

COPYRIGHT—W. B. KEEN, COOKE & CO., A.D. 1875.

